# A good patient? How notions of ‘a good patient’ affect patient-nurse relationships and ART adherence in Zimbabwe

**DOI:** 10.1186/s12879-015-1139-x

**Published:** 2015-09-30

**Authors:** Catherine Campbell, Kerry Scott, Morten Skovdal, Claudius Madanhire, Constance Nyamukapa, Simon Gregson

**Affiliations:** Department of Social Psychology, London School of Economics and Political Science, Houghton Street, WC2A 2AE London, UK; Department of International Health, Johns Hopkins Bloomberg School of Public Health, Baltimore, MD USA; Department of Public Health, University of Copenhagen, Copenhagen, Denmark; Biomedical Research and Training Institute, Harare, Zimbabwe; School of Public Health, Imperial College, London, UK and Biomedical Research and Training Institute, Harare, Zimbabwe

**Keywords:** Patient-provider relationships, ART adherence, HIV/AIDS, Social representation, Sub-Saharan Africa

## Abstract

**Background:**

While patient-provider interactions are commonly understood as mutually constructed relationships, the role of patient behaviour, participation in interactions, and characteristics, particularly ideals surrounding notions of ‘good’ and ‘bad’ patients, are under-examined. This article examines social representations of ‘a good patient’ and how these representations affect patient-healthcare provider relationships and antiretroviral treatment (ART) for people living with HIV.

**Methods:**

Using thematic network analysis, we examined interview and focus group transcripts involving 25 healthcare staff, 48 ART users, and 31 carers of HIV positive children, as well as field notes from over 100 h of ethnographic observation at health centres in rural Zimbabwe.

**Results:**

Characteristics of a good patient include obedience, patience, politeness, listening, enthusiasm for treatment, intelligence, physical cleanliness, honesty, gratitude and lifestyle adaptations (taking pills correctly and coming to the clinic when told). As healthcare workers may decide to punish patients who do not live up the ‘good patient persona’, many patients seek to perform within the confines of the ‘good patient persona’ to access good care and ensure continued access to ART.

**Discussion:**

The notion of a ‘good ART patient’ can have positive effects on patient health outcomes. It is one of the only arenas of the clinic experience that ART patients can influence in their favour. However, for people not conforming to the norms of the ‘good patient persona’, the productive and health-enabling patient-nurse relationship may break down and be detrimental to the patient.

**Conclusion:**

We conclude that policy makers need to take heed of the social representations that govern patient-nurse relationships and their role in facilitating or undermining ART adherence.

## Background

This paper examines how social representations of a ‘good patient’ affect patient-healthcare worker relationships and antiretroviral treatment (ART) in rural Zimbabwe. ART is a complex drug regime and requires repeated interactions between healthcare workers and patients over many years, during which issues such as diet, infections, reactions to antiretroviral drugs, drug adherence, and sexual practices must be navigated [1–5]. The relationships that develop between healthcare providers and patients, and the impact of social representations surrounding notions of good and bad patients, have not yet been examined in the context of ART. Patients negotiate their relationships with healthcare staff to enable them to access good service and ease their clinical experiences, primarily by seeking to perform the role of ‘good ART patient’. We suggest that these conceptualizations of appropriate patient behaviour and characteristics have several positive outcomes such as encouraging health promoting behaviour and easing potentially difficult health centre visits. However, some patients cannot conform to these notions, leading to a breakdown in the relationship between nurses and patients, which can undermine ART adherence.

Zimbabwe has suffered one of the most severe HIV and AIDS epidemics, with adult infection rates peaking at 26.5 % in 1997 but dropping to 14.3 % in 2009 [6]. ART scale up has enabled 56 % of adults and children in need of treatment (over 200,000 people) to access antiretroviral drugs (ibid.). While previously, clinical interactions focused on opportunistic infections and palliative care, ART enables nurse-patient relationships to develop surrounding the provision of life-prolonging drugs. ART requires repeated interactions between HIV positive people and healthcare staff over many years for check-ups, counselling and drug refill. This new medical regime presents new opportunities and challenges for the development of patient-healthcare provider relationships. Social representations surrounding ART users (i.e. collective values, beliefs and practices about ARV users, such as how they ought to behave and how they ought to be treated) are being developed throughout Africa in response to the increasing availability of antiretroviral drugs. This new era in HIV care affords a unique opportunity to examine the development of symbols and representations in medical interactions. More directly, it is vital to explore how representations of good patients are helping and hindering access and adherence to a lifesaving treatment.

In this paper, we first outline existing academic literature on patient-healthcare provider interactions, highlighting the need for research on the construction and response to notions of ‘good’ patients, and situating our work in the theoretical arena of social interactionism and performativity. We then present our study of notions of ‘good’ patients in the context of antiretroviral treatment provision in rural Zimbabwe.

### Patient-provider interactions

There is extensive literature on patient-healthcare provider interaction [7]. Positive clinical experiences, particularly the development of good quality relationships between healthcare workers and patients, has been found to promote ART adherence [8]. While patient-provider interactions are commonly understood as mutually constructed relationships [9], the role of patient behaviour, participation in interactions, and characteristics, particularly ideals surrounding notions of good and bad patients, are under-examined. Research on patient-healthcare provider interaction tends to focus on the influence of healthcare provider characteristics, such as levels of job satisfaction and frustration [10], biases (e.g. [11]) and technical or communicative skill competence, on healthcare interactions (e.g. [12]), with little examination of patient perspectives. Fleischer, Berg et al. [7:339] conclude in their systematic review of literature on the nurse-patient interaction that the “involvement of patients and their role in communication often is neglected by authors”.

### ‘Difficult’ or ‘bad’ patients

Notions of ‘difficult’ patients are invoked in studies of mental health care and, less frequently, care for patients with physical illnesses [13]. Research on ‘difficult’ patients is usually from the perspective of what healthcare workers think is difficult or bad and the worker’s capacity to cope. For instance Sharpe, Mayou et al. [14] found that doctors classified difficult patients as those with medically unexplained symptoms; co-existing social problems; and severe untreatable illness; the healthcare providers in a study by Elder, Ricer et al. [15] on dealing with difficult patients classified them as the ‘worried well’, social visitors, and patients who were non-compliant, non-appreciative, drug seeking, and those having multiple medical complaints. ‘Bad’ patients can also be those with uninteresting medical conditions or those seen to have been morally deviant and as such deserving of their illness [16, 17]. Moral judgement and medical professionals’ belief in deservingness has been especially explored in relations to HIV [18]. Healthcare worker perceptions and coping is emphasized with little discussion of the role of patients and the social context of hospitals, communities and beyond, in constructing and responding to ideals of patient behaviour.

### ‘Good’ patients

‘Difficult’ and ‘bad’ patients can only be understood in contrast to ‘good’ patients, a commonly invoked concept in the media and the grey literature (e.g. [19]). For example, an article written by a physician for the *Time* magazine describes “a good patient” as someone who listens, follows directions, asks relevant questions, shows trust in his or her doctor and ‘massages’ the doctor’s ego [20]. The academic literature on the construction of notions of good patients, how patients and staff respond to these notions, and how these notions affect interactions and treatment outcomes is more limited. McCreaddie and Wiggins [21] discuss the ‘good patient persona’ as one who demonstrates willingness to comply, being over-grateful for services, and being seen as coping positively. Wortman and Dunkel-Schetter [22] and Taylor [23] identify being silent, passive and accepting as the perceived role of a ‘good patient’. Recent research on notions of ‘good patients’ in resource-poor settings suggests that South African nurses see ‘good’ patients as those who are friendly and calm most of the time, accept help without complaining and are very polite [24]. This paper seeks to expand on the notion of ‘good patients’, by examining how patients and nurses construct and invoke notions of ‘good patients’ and how these notions affect interactions and treatment. As mentioned above, ART is a key area for investigating social representations of patient behaviour because it requires repeated interactions between ART users and health workers over many years.

### Theoretical framework

Social representation theory (cf. [25]) is the central lens through which the notion of good patients, in the context of ART provision in rural Zimbabwe, is examined. Social representations are the shared knowledge that enables a group of people to make sense of their shared social world and to communicate about it [26]. These representations of the world are dynamic and can thus be reinforced or re-constructed through social interactions that reproduce traditional understandings of the world or enact new ways of seeing the world and acting within it.

These interactions, which reinforce or reconstruct, can be understood through the theories of symbolic interactionism [27] and performativity [28], which emphasise the role of micro social interactions and everyday performances in responding to, creating and modifying meaning. Drawing from Foucault, particularly his concept of the ‘meticulous rituals’ of power [29], Butler described performativity as “…that reiterative power of discourse to produce the phenomena that it regulates and constrains” (Butler quoted in [30]). It is through everyday, minute social interactions that humans entrench or challenge power and meaning. Symbolic interactionism holds that people interact by interpreting and ascribing meaning to one another’s actions – rather than reacting to some sort of innate meaning. In this way, people communicate through socially constructed symbols. We will apply these theories to notions of ‘good’ and ‘bad’ patients, exploring how patients and healthcare workers must continuously relate to socially constructed notions of patient/provider behaviour as they participate – and perform – within the parameters of daily clinical interactions.

## Methods

The study is guided by the following research question: In what ways do social representations of a ‘good patient’ govern nurse-patient relationships and facilitate/hinder ART adherence? To answer this question we draw on a mixed-method and multi-site qualitative research design, enabling an exploration of the various environments in which social representations are constructed and responded to. Ethical approval for the study was granted by the Medical Research Council of Zimbabwe (Ref: A/681) and the Imperial College Research Ethics Committee (Ref: ICREC_9_3_13). Written informed consent for participation in the study was sought and granted from all participants. Names and details of the health clinics have been anonymized to protect the identity of participants.

### Study setup and sampling

The study took place in the Manicaland province of eastern Zimbabwe where the HIV prevalence is approximately 20 % [31]. Residents in this area are primarily subsistence farmers living in rural homesteads, often without electricity or plumbing. Many families have members working in urban centres or for large commercial farming and forestry estates, some of whom send money back to their rural home. Poverty is a major challenge and many local people receive food aid from international organizations. Health facilities in the region deal with frequent staff, equipment and electricity shortages. It was against this background that we sampled a range of micro-contexts within rural Zimbabwe (a small clinic, a large government hospital and a large Anglican hospital) in order to identify the social representations that arose throughout the region rather than issues specific to any one health centre or participant. Since the research was focused on collectively developed and enacted representations, we report findings that resonated at all sites and do not distinguish between the three health centres.

Using purposeful sampling, we recruited a total of 104 participants (48 adult ARV users, 31 carers of children on ART and 25 health staff). Carers of children on ART were included because they interact with healthcare staff on an on-going basis just like ART patients and must similarly navigate the clinic space to access services. They generally relate to health centre staff in much the same way as patients. For example, carers with children on ART wait in the same queues as adult ART patients and interact with the same staff. Most ART patient/carer participants were recruited through openly HIV-positive community members known to the researchers through previous HIV/AIDS research. Others were approached as they visited hospital or clinic sites. A few participants approached the researchers asking to be interviewed because they had heard about the project. Researchers’ requests to interview a person on ART were only refused in one case, by someone who cited time limitations. Healthcare staff were primarily nurses but also included four HIV counsellors, a nurse aid, a pharmacist technician and an administrative clerk. Staff members had often been born in the region or had moved there through marriage, sharing a common identity with patients. Researchers received permission to conduct the research from each health centre’s doctor or nurse-in-charge. Staff members were then approached individually to participate; all agreed.

The nurses ranged in age from 29 to 57 years (average age of 41). The other health centre staff were all in their 30s. The carers ranged in age from 39 to 79 (average age of 55) and were caring for HIV positive children ranging in age from 4 to 16 years; many were the respondents’ children or grandchildren but some carers looked after nieces, nephews and more distant relatives. The adult ART users ranged in age from 30 to 65 (average age 45).

### Data collection and analysis

To unpack the symbolic resources that are likely to play a key role in shaping patient-healthcare provider interactions, we set out to examine the perceptions, experiences, practices and behaviours of health service users and providers through i) in-depth interviews, ii) focus group discussions (FGDs) and iii) ethnographic observation in clinics. We conducted 14 interviews and 4 FGDs with adult ART users, 12 interviews and 3 FGDs with carers for children on ART, and 18 interviews and one FGD with health staff (see Table 1). The FGD with health centre staff involved five nurses and two HIV counsellors.

**Table 1:** Summary of study participants

	Participants			Interviews			FGD
	Male	Female	Total	Male	Female	Total	(Mixed gender)
Health centre staff	13	12	25	9	9	18	1
Nurse	9	9	18	6	7	13	-
Other staff	4	3	7	3	2	5	-
Carer of child	2	29	31	0	12	12	3
Adult ARV user	16	32	48	8	6	14	4
Total	31	73	104	17	27	44	8

The interviews and FGDs were conducted using topic guides that explored areas such as social support and ways of coping with HIV and ART, issues surrounding treatment adherence, and experiences at the health care centre. To gather explanations of social representations of ‘good’ and ‘bad’ ART patients, participants were asked: What makes a ‘good ART patient’? Patients were asked ‘How can you tell if the nurse thinks you are a good/bad patient?’ and ‘What do nurses think is a good/bad patient?’ During focus groups with patients, participants were invited to perform a role play of ‘a good day at the clinic’ and ‘a bad day at the clinic’. These role-plays revealed a great deal about how ART patients perceive appropriate and inappropriate clinical interactions.

In addition, over 100 h of ethnographic observation were conducted at the health centres, observing interactions as HIV-patients waited for the doctor, paid hospital fees, visited the pharmacy, and waited for nurses to review their progress on ART and prescribe refills of their ARVs. Researchers did not observe private interactions between patients and staff. Observation focused on hospital activity, including interactions between patients and staff and the arrangement of people in hospital spaces. As with the FGDs, our observations allowed us to explore interactions between people and within social contexts where meaning is collectively constructed. Data were collected by three Shona-speaking fieldworkers (two female and one male) and a fourth researcher (female) working with the male Shona-speaking fieldworker. The researchers came from a research centre in the capital city Harare and were trained to conduct qualitative research. All audio files were translated into English and transcribed by trained researchers. To thank the informants, FGD participants were given soap, and interviewees were given a t-shirt.

The data were analysed using Attride-Stirling’s [32] thematic network analysis. As social representations are collectively negotiated properties of social groups rather than individuals, our unit of analysis was the data corpus as a whole. We therefore uploaded all interview and FGD transcripts, as well as extensive ethnographic notes (over 60,000 words in total) into Atlas.Ti, a qualitative analysis software package. This also enabled us to triangulate emerging themes and discuss disagreements within the data.

Following preliminary readings and re-readings of the transcripts, two researchers began coding the transcripts, inductively generating a total of 225 ‘basic themes’ (segments of text classified within interpretative headings and linked to informant identities as patients, carers or nurses). As we are not seeking to report on all of these codes, we identified codes and segments of text relating to representations of ART patients, including but not limited to specific references to characteristics and behaviours of ART patients or those who choose to seek (or avoid) HIV testing, counselling and treatment, for detailed re-reading and re-coding if necessary. Through this process we identified 27 basic themes, covering 16 issues that we believe deepen our understanding of how the social representations of a ‘good patient’ that govern nurse-patient relationships can facilitate/hinder ART adherence. To lift these basic themes into higher order themes, we clustered the basic themes within wider ‘organising themes’, which through progressive re-examination and refinement were gradually ‘reduced’ to a single ‘global theme’ (see Table 2) – giving rise to the topic of this paper. The four organising themes that arose from our analysis relate to: i) nurse power, patient vulnerability, ii) distressing situations patients seek to avoid, iii) predictably unpredictable clinic visits and finally iv) representations of a ‘good patient’ and why patients seek to perform within them. These organising themes provide the sub-headings in the findings section below.

**Table 2:** Thematic network: from codes to global theme

Basic Themes (codes)	Issues discussed within basic themes	Organising themes	Global Theme
- Nurses police adherence	• It is the nurses responsibility to monitor adherence.	1. Nurse power, patient vulnerability	Social representations of a ‘good patient’ that govern nurse-patient relationships and facilitate/undermine ART adherence
- Nurses are powerful	• Nurses are in a powerful position compared to rural, poor and uneducated patients.		
- Patients powerless			
- Nurses are frightening			
	• ARV users are not in a position to assert their needs or dissatisfaction.		
- Nurses reprimand	• Nurses are powerful mediators between doctor and patients.	2. Distressing situations patients seek to avoid	
- Young nurses			
	• Young nurses appear more authoritarian.		
- Nurses treatment power			
	• Nurses have the power to undermine doctors.		
- Punishments	• Nurses can decide to punish patients if they do not behave as expected.		
- Nurse expectations			
- Clinic visits	• Patients do not know what their monthly clinic visit will be like	3. Predictably unpredictable clinic visits	
- Worry			
- Waiting time			
- Unpredictability	• Patients often have to wait for a long time to be seen.		
	• Patients try to make the best out of their visits to the clinic.		
- Well behaved	• ARV users should comply with the instructions given, including attend timely review dates	4. Representations of a ‘good patient’ and why patients seek to perform within them.	
- Follow instructions			
- Obedient			
- Timely review dates			
- Is taking drugs timely	• ARV users should be obedient to nurses		
- Being positive			
- Being good natured	• ARV users should be positive and content with the service received		
- Wait patiently			
- Honesty			
- Listening closely			
- Committed to treatment	• ARV users should be open, honest and accept help readily		
- Clean and smart	• ARV users have sourced out what nurses would like from them and maintain a good relationship by living up this.		
- Negotiating a good relationship			
- ‘Good patients’ do better			
	• ARV users should be clean and well dressed		

### Findings

Patients and healthcare providers easily referenced notions of good and bad patients and overwhelmingly agreed on these characteristics, suggesting that understandings of appropriate ART patient behaviour are well developed and tightly defined. To gain a better understanding of the processes and representations that govern such understandings, we first outline the context in which these representations are constructed, namely the authority and power of healthcare workers, the emotional distress that patients can face at the clinic and the uncertainty of many aspects of each clinic visit for patients. We suggest that trying to be a ‘good patient’ is one of the only arenas of the clinic experience that ART patients can influence in their favour. We then describe the characteristics of ‘a good patient’ and discuss how patients try to perform within the parameters of the good patient persona in order to secure the most favourable and smooth treatment possible.

#### Nurse power, patient vulnerability

Nurses are in a hugely powerful position compared to their rural, poor and often less educated patients. Their power is clear in many ways; they wear uniforms, which signal their biomedical authority, reprimand patients when they fail to follow directions, have control over queues and order of access to service, and are responsible for administering drugs.

When nurses are unhappy with a patient, they can cause significant emotional distress for patients. A carer of a child on ART said: “If there is a rude nurse on duty I get weak and say ‘better stay at home because I cannot go to face that rudeness’” (female, 37, caring for her two HIV positive children). The fear nurses can instill in patients and carers of child patients may lead them to delay going to the clinic for as long as possible, undermining ART adherence:

They should at least have some courtesy or talk to the patients properly, because you end up being afraid of going back there. If a problem arises and you think of going to [the hospital], you think twice. You think of the nurses that have once ill-treated you and you are hesitant. That’s when you keep on looking at the patient without taking her to hospital to the extent that the patient may even die. (female, 40, caring for 7-year-old niece)

Staff who are not pleased with patients can act negatively towards them with relative impunity and patients have no power to assert their needs or dissatisfaction. A 59-year-old woman caring for her 16-year-old granddaughter explains “Even if you see them doing the wrong thing, what will you say? Nothing.” And another says “If you try to complain they might even shout at you” (patient, female, 42). In another instance, when the interviewer probes a respondent further about addressing issues with nurses, the respondent (female, 59, caring for 16-year-old granddaughter) says:

*Respondent*: But we can’t go there and tell them.

*Interviewer*: Why not, do you know that it is your right to say out what you feel?

*Respondent*: Oh my goodness and you will still want to go back there?

*Interviewer*: Yes, you can go back, you can see the senior nurse or the doctor, you are free to tell him that you were not happy with the way you were treated.

*Respondent*: We do not even know about that… We will be afraid. (Female, 59, caring for 16-year-old granddaughter)

#### Distressing situations patients seek to avoid

Nurses can exert their power in ways that result in difficult situations for patients. In this section, we discuss the types of negative situations that can occur when nurses are not pleased. It is vital to understand the ways in which patients can be poorly treated to understand their desire to smooth interactions as much as possible, and thus their efforts to perform as ‘good patients.’

The most commonly referenced emotionally distressing situation was harsh reprimands by nurses. Sometimes these were seemingly unprovoked, as in the following quotation in which a patient describes being told to line up and sit down in a harsh manner while waiting for service:

… He will be shouting different kind of instructions, for example: “make sure you are in line” and “may everyone sit down, I won’t serve anyone standing up”. The benches will be full so some will sit on the floor…if you try to complain he might even shout at you (Patient, female, 42)

Reprimands were often linked to ART-related transgressions, such as imperfect adherence or failure to come to the clinic at the correct time. Nurses are often understanding about the difficulties patients face on ART and in many cases respond to transgressions in a calm way. However, there are enough exceptions to such compassionate responses that every visit brings the possibility of being reprimanded harshly.

I am sure the nurses and doctors get so frustrated when they look at the card and see how somebody has been defaulting or just not coming on their review dates. They get so frustrated and at times we would feel that we were ill-treated but we would have also caused that treatment on ourselves. (Patient, female, 50)

Younger nurses and student nurses, who have less power in relation to other staff but significant power over patients, were often cited by patients as the most likely to speak harshly.

Maybe I will go and join the queue of those waiting for wounds medication or eye medication, then they will come (student nurses) and harass you, ‘old woman why are you seated there, why do you go and sit there, is that place the rightful place for you to sit?’. You try to answer politely, I didn’t know, thank you for telling me.’ That’s the nature of these students. (Female, 40, caring for 7-year-old-neice)

As the following quotation illustrates, nurses have the power to undermine the doctor’s choice of drug prescription—or at least threaten to. This does not apply to the ARVs themselves, but to medicine for opportunistic infections that occur in HIV-positive people.

The nurses make it appear like this doctor is just prescribing drugs for us that we don’t deserve, because when we see the doctor and he prescribe some drugs the nurses will complain and make it appear like the doctor is doing things that seem like he is just favouring us. There are times when we would avoid saying we have any other illnesses for fear that if the doctor prescribe any other medicines the nurses will complain before they give you. It appears like they don’t agree with what the doctor would have done. I think this doctor should just bring medicines so that whatever he prescribes he will also dispense without us having to go see the nurse when we go for our reviews. It’s something that is so worrying. (Patient, female, 42)

In the above quotation, the patient suggests that she and her peers were not seen as deserving of the prescription and thus faced the nurses’ complaints and threats to withhold the medicine. Other emotionally distressing situations created by nurses for some patients were seen during observation. A patient who could not pay the US$1 consultation fee was sent from department to department and ultimately told to wait until everyone else had been served to see if there was time for him at the end. At another time, when several patients were crowding around the door of the nurse consultation room, they were told to get back into a quiet line, and that the nurses would stop serving everyone if the patients did not behave in an orderly fashion.

It is evident that, in the health clinics, it is the nurses who are in control. They have the power to reprimand patients strongly for transgressions, treat patients unkindly, and make the clinic visit more stressful and difficult than it needs to be in many other ways. Likewise, they are able to be extremely friendly, helpful and encouraging to patients. Patients can seek to gain the favour of nurses, keep them feeling happy, and avoiding irritating them, in order to smooth their clinic experiences.

#### Predictably unpredictable clinic visits

Clinic visits are very unpredictable. These range from easy visits taking less than two hours to prolonged full day odysseys through multiple queues and interactions. On many clinic visits, patients simply do not know what is going on, whether there will be enough time for the nurses to see them and whether they will receive their pills. During our observations, there were times when patients who arrived at 5 am spent all day in various queues, leaving only at closing time. Sometimes, having not received the service they needed - usually to see the doctor in order to begin or adjust their ART - patients slept over at bus stands or on hospital cots in order to try again the next day. On good days, patients arrived at 8 am and by 9:30 am were on their way home, having been checked by nurses and given their pills at the pharmacy. On busy days, the queue of over 30 waiting patients sometimes moved at a rate of about one patient an hour but, in the last hour before 4 pm closing, would speed up dramatically to process the remaining 25 patients. Patients in the queue spend much of the day worrying whether they would be seen by the nurses in time to go to the pharmacy. On a day when the ARV delivery was delayed, patients, with no explanation, waited for six hours. Towards the end they began comparing how many days’ worth of supplies they had left, saying “What will we do if he [the doctor who brings the pills] doesn’t come?” and “We will die because we can’t have supplies if he is not around.” The seriousness with which patients take their treatment and the extremely powerful position of nurses to ease or aggravate the stress and discomfort associated with these visits makes positive relationships with staff of central importance to ART patients.

In the context of the asymmetry of power between patients and nurses, the capacity of nurses to create emotionally distressing situations, and the unpredictability surrounding key aspects of the clinic visit, patients have strong incentives to try to positively influence their clinic experiences through using what little they can control: their own behaviour towards nurses. This unequal relationship, and the dependency of ARV users on nurses, gives rise to a number of representations of a ‘good patient persona’. Patients can seek to reduce the emotional distress of clinic visits through signaling to nurses that they are ‘good patients’, and thus seek to avoid being reprimanded and treated roughly. In the next section, we will examine what these representations of ‘good patients’ are, and how patients perform within these representations during interactions with nurses.

#### Representations of a ‘good patient’ and why patients seek to perform within them

Patients are eager, and sometimes desperate, to perform as ‘good’ ART patients to ease their clinical interactions.

The social representation of a good ART patient includes being obedient to the nurses’ instructions, enthusiastic, patient about waiting in queues, and open. In the following quotation, a nurse explains her perception of a good patient, emphasizing the value of following directions, waiting quietly in the queue and being cheerful:

There are patients who follow what we encourage and they take their pills on time, they also don’t miss their review dates and when we calculate the adherence rate you would find it will be 100 %. These patients would not miss their review dates and they don’t put a lot of pressure on nurses to be given preference to be served first. They wait in the queue and when their turn comes they come in and they will be cheerful and one can see that such patients recover better than those who default. (Nurse, female, 29)

Following nurse instructions is a vital way to signal to staff that you are a good patient in order to secure ‘good patient’ treatment. A male patient (age 37) states succinctly: “People who get the best treatment are those who follow the instructions that they would have been given.” The following quotations provide further explanations of obedience as a key element of the social representation of ‘good patients’.

A good patient will never miss their appointments or even default on their time of taking pills because they understand why they are taking those pills and they also don’t want to spoil their relationship with us. (Nurse, male, 46)

I also want to say if you follow what the nurses say, they will treat you as a good patient but those who don’t listen to what they say are considered difficult patients. They would think those are just trying to create problems for the nurses. (Patient, female, 42)

When you are given a review date, you should keep the date, failure to do that that’s when you will cross their path […] For you to remain in good track, then follow the nurses’ instructions (Female, 38, caring for 4-year-old daughter)

Good ART patients accept help readily and are honest and open. A male pharmacist technician (age 36) explains “We see them every month and serving them is a pleasure because they are always cheerful and they ask where they do not understand.” This sentiment is echoed frequently by nurses and patients. For example, a female patient (age 35) explains that “nurses want a patient who explains clearly what they would be going through not a person who hides what they are suffering from.” The following comments from nurses expand on the importance of talking freely and openly:

I really feel satisfied with the patients when they are open and truthful… particularly in relation to compliance. They might tell you that one day they delayed taking their pills by about 30 min. They could easily decide not to tell me this, but they have understood that they have to be honest (Nurse, female, 56)

What we call a good patient is that one who is willing to learn and also asks where they don’t understand. There are patients who say ok, I understand yet they would have not understood anything… So the one who asks questions and also listens carefully and does what we recommend, then that’s a good patient. I really prefer patients who are open to those who are just quiet.... Those who are free and open will always make our job easier. I would really feel that this is a good patient who is keen to understand (Nurse, male, 56)

Cleanliness and neat dress were cited as further traits of ‘good’ patients, with patients focusing on this point more than nurses. For example, after we gave participants in focus groups soap, as a token of gratitude for participating, a male patient (age 53) said: “We also want to thank you for the soaps that you have brought us. I think we will keep ourselves smart when we visit the [ART] clinic.” At other times, patients explained that “nurses want to serve patients who are smart [i.e. well turned out]” (male, 58) and that “We have learned what these nurses want. Nurses do not want people who are dirty and who don’t bathe” (female, 50).

Patients seek to nurture a positive relationship with the clinic staff by performing many clear displays of ‘the good patient persona’ and trying to avoid doing anything to incur the irritation of staff. Enthusiasm and gratitude for treatment was demonstrated by jumping up immediately when called into the nurse’s consultation room, initiating greetings with staff, and thanking staff profusely for their help – as well as by not mentioning any dissatisfaction. Even when waiting in very long queues for many hours patients, patients were strategic in communicating their frustration with the nurses to one another and not to staff.

When patients expressed irritation, it was often directed at one another, usually in the form of harsh exchanges about the order of the line-up, or spoken in ways that ensured staff did not hear them. For example, one morning at the government hospital, the two nurses serving ART patients went for their usual 11 am tea break, leaving about 40 people in line and having served less than five. Two male patients in line, who had been waiting for approximately four hours, asked angrily, “When are you coming back?” and “Why are you both going at the same time?” but were careful to speak only loud enough for their peers in line to hear but too quietly for the nurses to hear. In fact, almost every expression of dissatisfaction (always linked to slow service) was carefully spoken out of hearing range of the staff, including another male patient saying, in response to a two hour wait at the pharmacy for pills, “If I was at a traditional healer’s I would have been served a long time back”.

When patients come from their monthly review date, they bring any remaining drugs which the nurses count as a way of checking the patient has adhered to the treatment. In the following quotation, a nurse explains how patients explain poor adherence proactively, and, in doing so, highlights patient efforts to appear ‘good’ (and avoid being reprimanded) in the face of wrongdoing:

…as we will be counting the tablets, we will always tell them that we are not on a fault finding mission but that we just want to help one another. So every time they tell you before you even ask them, ‘Nurse my tablets do not tally, I lost some, I took some from the bottle and pocketed them going where I was going and they got washed up in the pocket.’ They say everything, before you go any further. Some will even tell you that somebody borrowed some from me. (Nurse, female, 34)

The quotation illustrates patients’ efforts to appear enthusiastic and committed to treatment when they have transgressed. In an effort to appear open and honest, especially in relation to less-than-perfect adherence, patients offer explanations to nurses before even being asked. This not only highlights the authority and power of nurses in their ART treatment, but also the fear patients have of doing something wrong and being reprimanded and how they carefully navigate their interactions with the nurses.

## Discussion

Our research sought to explore how both patient and healthcare provider perceive and respond to representations of ‘a good patient’. The theory of symbolic interactionism highlights that all people, including patients and nurses, communicate through a series of socially constructed symbols. Our research identified many ways in which patients ‘signal’ their goodness and deservingness of treatment or their respect for the medical establishment to their nurses and other healthcare givers. Performing within the ‘good patient persona’ is seen to be a way patients can influence their hospital experience by increasing their chance of receiving good services. Securing good care is particularly important in resource-poor settings where staff-time and medication are in short supply. Patients have very little control over the central aspects of the clinical experience such as whether they will access the care or drugs they need. However, they can attempt to control one aspect of the clinical visit: the nurses’ perception of them, which has a significant influence on how smooth and pleasant the visit will be. By carefully understanding and performing exactly what nurses want, patients can avoid irritating nurses (which can lead to lectures, yelling, and sometimes slower service) and can to try to remain on the nurses’ good sides (which maximizes pleasant interactions and support).

Several key characteristics of ‘good’ and ‘bad’ patients that were identified by healthcare providers and patients in this study align with those reported in the existing literature. McCreaddie and Wiggins [21] and Elder and Ricer et al. [15] highlighted compliance and gratitude, two main characterizations that patients in our study sought to embody. As pointed out in the literature review, there is a dearth of patient input in research on notions of good and bad patient behaviour. By adding patient views, we discovered many of the same social representations of ‘good patients’ that were determined by research seeking only healthcare worker opinions. This confirmation of previous research suggests that healthcare workers, because of their elevated position of power, exert a strong guiding influence on social representations of good patients to fit their needs. However, patient input illuminated some features that have not previously been discussed. For instance, patients emphasised the importance of patient appearance (in terms of cleanliness and smart dress) in pleasing healthcare staff—perhaps exposing a component of ‘the good patient persona’ that healthcare workers chose not to discuss with researchers or were not aware that patients perceived them to care about.

Unlike the findings of Murcott [17] and McCann [18], moral judgement about how a patient contracted HIV did not seem to play a role in patient capacity to be seen by staff as a ‘good patient’. In addition, there was little evidence that staff applied moral judgement to how HIV-positive ART patients behave. Instead of framing ART adherence as a moral duty or an act of social responsibility—good ART adherence does keep viral levels low thus reducing the chances of infecting others—staff primarily discussed ART adherence as vital for the continued health of the patient.

Most of the characteristics of a good ART patient are things that will help interactions at the hospital go smoothly and increase positive health outcomes. In many ways, acting as 'a good ART patient' benefits both patients and nurses because it means patients try hard to take their drugs perfectly and are friendly to healthcare staff struggling to scale up ART services in extremely challenging settings. Nurses already struggle with resource shortages, payment interruptions and lack of opportunities for advancement. Patients who are irritated by slow service and may want to express this anger are careful not to take it out on nurses—a good outcome for the most part because, in many cases, it is unlikely that the nurses ought to bear the brunt of this frustration.

Despite the potentially beneficial outcomes of the social representation of ‘good ART patients’, there are some negative potential outcomes that requires further attention. Patients who cannot perform within the ‘good patient persona’ must endure very trying and exhausting visits to the clinic. There are many reasons why patients cannot exhibit the signs of being a good patient, and these patients bear the brunt of nurse reprimands, which reduces quality of life and may discourage retuning to the clinic. For instance, patients can be overwhelmed by the clinical environment and the lifelong requirements of ART, which leads some to poorer adherence. Rather than being treated harshly, these patients would benefit from further support and counselling.

There are several limitations to this study. Though we were able to recruit a large number of individuals, it is likely that some responses may have been influenced by social desirability bias. However, our mix of methods and respondents helped minimize this risk. As with any qualitative study, our findings may not generalize to other settings, as our participants, geographic and cultural environment may vary from others. Finally, despite the consistency in our findings, not all the socially constructed symbols and signals identified to govern patient-healthcare provider interactions and ART adherence may apply to the same degree across clinics and communities.

## Conclusion

In this article, we examined how notions of a ‘good patient’ affect patient-healthcare provider relationships and antiretroviral treatment. Both patients and providers have very clear and similar understandings of appropriate patient behaviour and characteristics. Characteristics of a good patient include obedience, patience, listening, enthusiasm for treatment, physical cleanliness, honesty and correct adherence. ‘Bad patients’ are those who do not listen, do not commit to treatment, fail to come to the clinic on the correct appointment days, are not clean or smartly dressed, do not pay for treatment and are impatient or impolite. We suggest that many characteristics of a ‘good patient’ promote positive outcomes from HIV-positive people on ART. These positive outcomes include smooth interactions at the clinic in the context of severe resource limitations that lead to shortages and delays medical health through encouraging ART adherence and lifestyles that support healthy living with HIV. However, it is not always possible for patients to perform within the characteristics of a ‘good patient’. For these patients, clinic visits can be highly stressful and discouraging, leading some patients to avoid the clinic entirely.

This research focused on notions of good patient behaviour as it pertains to ART patients in Zimbabwe where ART had only been widely available for about one year prior to the research period. ART is an on-going regime of care, necessitating the development of nurse-patient relationships over many years. Further research into these relationships over the long term would increase our understanding of the construction of social norms surrounding patient-provider interactions and the effect of these norms of medical care. For instance, as ART becomes a well-established regime, healthcare workers may become less focused on or concerned with patient adherence. Furthermore, will patients remain passive and satisfied with the unpredictable and occasionally abusive relationships for many years or will a greater sense of assertiveness and entitlement develop? On-going research into these areas would help generate a better understanding of social representations of good patients and the ways that behavioural norms and power dynamics change over time. This research will particularly contribute to our understanding of chronic patient care in resource poor settings, where to date the vast majority of patient-provider interaction revolves around one-off acute care.

An additional point that would warrant further research is the relationship between ‘good’ and ‘bad’ patient characteristics and gender norms in the area. It seems that notions of ‘good patients’ often go against hegemonic notions of masculinity in Zimbabwe [3]. Male identity is Zimbabwe is often linked to active rather than passive roles, avoiding healthcare centres rather than coming on a regular basis and dominant rather than submissive roles. Nurses lamented the fact that far fewer men than women come to the clinic for HIV testing and ART. Perhaps they avoid clinics, in part, because they find it difficult to perform within the boundaries of the ‘good patient persona’ and thus receive brusque, off-putting treatment.
